# Electronic Tongue Recognition with Feature Specificity Enhancement

**DOI:** 10.3390/s20030772

**Published:** 2020-01-31

**Authors:** Tao Liu, Yanbing Chen, Dongqi Li, Tao Yang, Jianhua Cao

**Affiliations:** School of Microelectronics and Communication Engineering, Chongqing University, No. 174 Shazheng Street, Shapingba District, Chongqing 400044, China; yanbingchen@cqu.edu.cn (Y.C.); dongqili@cqu.edu.cn (D.L.); 20161213071@cqu.edu.cn (T.Y.); jianhuacao@cqu.edu.cn (J.C.)

**Keywords:** electronic tongue, feature extraction, kernel extreme learning machine, specificity enhancement

## Abstract

As a kind of intelligent instrument, an electronic tongue (E-tongue) realizes liquid analysis with an electrode-sensor array and certain machine learning methods. The large amplitude pulse voltammetry (LAPV) is a regular E-tongue type that prefers to collect a large amount of response data at a high sampling frequency within a short time. Therefore, a fast and effective feature extraction method is necessary for machine learning methods. Considering the fact that massive common-mode components (high correlated signals) in the sensor-array responses would depress the recognition performance of the machine learning models, we have proposed an alternative feature extraction method named feature specificity enhancement (FSE) for feature specificity enhancement and feature dimension reduction. The proposed FSE method highlights the specificity signals by eliminating the common mode signals on paired sensor responses. Meanwhile, the radial basis function is utilized to project the original features into a nonlinear space. Furthermore, we selected the kernel extreme learning machine (KELM) as the recognition part owing to its fast speed and excellent flexibility. Two datasets from LAPV E-tongues have been adopted for the evaluation of the machine-learning models. One is collected by a designed E-tongue for beverage identification and the other one is a public benchmark. For performance comparison, we introduced several machine-learning models consisting of different combinations of feature extraction and recognition methods. The experimental results show that the proposed FSE coupled with KELM demonstrates obvious superiority to other models in accuracy, time consumption and memory cost. Additionally, low parameter sensitivity of the proposed model has been demonstrated as well.

## 1. Introduction

Electronic tongues (E-tongues) consist of an electrode-sensor array and a machine-learning unit, and have made considerable contributions to liquid analysis since 1990s [1,2,3]. The adopted electrode sensors for an E-tongue often have both a low selectivity and high cross-sensitivity while the machine-learning part realizes smart identifications according to the responses of the electrode-sensor array [4]. As far as we know, E-tongue systems have been applied in extensive scenarios such as environmental monitoring [5,6], food identification [7,8,9] and beverage discrimination [10]. With regard to beverage discrimination, several kinds of beverage, including coffee [11], tea [12,13], wine [14,15] and dairy products [16], have been assessed by E-tongues to clarify their origins, grades or brands. Among these studies, tea and wine seem to have been used as the main testing substances.

Current E-tongues can be broadly divided into three categories according to the employed sensors: potentiometric [17,18], optical [19,20] and voltammetric types [21,22]. The potentiometric E-tongue usually operates on the basis of high impedance sensitive membrane sensors. It relies on a working electrode and a reference electrode to constitute a two-electrode system [23]. The working electrode potential varies according to the nature and strength of the solutions, which reflects the characteristics of the samples. Different from potentiometric methods, optical E-tongues involve various optical sensors that consist of three main parts—a light source, an optrode and a detector [24]. The optical properties of the indicator contained in the optrode would be changed when the sensor is exposed to a target analyte, and the detector can monitor this change and convert the optical signal into an electrical one. Conversely, the voltammetric type E-tongue is based on noble metal bare electrodes, and it employs a three-electrode system for measurement, containing a working electrode, an auxiliary electrode and a reference electrode [25]. The potential of the reference electrode is assumed to be constant while a signal loop is formed between the auxiliary and working electrodes for response signal obtainment. Among these three kinds of E-tongue, the voltammetric type has drawn more attention owing to its abundant original responses, high signal-to-noise ratio, simple operation and low cost. Winquist et al. reported a voltammetric E-tongue with an array of noble-metal electrodes which sensed the characteristics of liquid substances and compounds in a large amplitude pulse voltammetry (LAPV) manner [26]. The collected LAPV response is regularly a digital time series containing huge amount of data due to the high sampling rates of the devices. In other words, the raw responses of a LAPV E-tongue are high-dimensional vectors, which may include any redundant features for following recognitions. Thus, a fast and effective feature extraction method is needed to obtain concise and meaningful features from raw responses. Consequently, dimension reduction methods have been utilized to extract features from original responses. Primarily, Principal Component Analysis (PCA) has been widely used as a classic feature extraction method with satisfied signal compression ability for LAPV responses in recent studies [27,28,29]. Furthermore, the concealed information in the LAPV time series could be revealed by time-frequency-analysis methods. Palit et al. employed discrete wavelet transform (DWT) to extract features from E-tongue responses [30]. However, the above methods directly process the original electrode responses, and may ignore the common-mode signals existing in different electrodes. Here, the “common-mode” signals mean the high-correlated parts in different working-sensor responses caused by the same LAPV excitation signal. The amplitude of the common-mode signal regularly occupies most of the raw LAPV responses, which may cover up the specificity of different sensor responses. As a result, the recognition performance of a LAPV E-tongue would be degraded unavoidably with common-mode signals.

In this paper, the electrode sensor response is regarded as a superposition of specificity and common-mode components. We consider the specificity parts as a significant basis for E-tongue classifications, whereas the common-mode components in different sensor responses provide harmful information for recognition. According to the above assumption, we have proposed a feature enhancement method using a nonlinear specificity metric that alleviates the common-mode components in sensor responses. Meanwhile, we introduce a machine-learning model that integrates the proposed feature enhancement method and kernel extreme learning machine (KELM) for E-tongue recognition. The main work of this paper can be summarized as follows:

(1) A specificity metric scheme is presented to enhance the useful features in electrode responses in Hilbert space by exploiting a kernel function.
(2) We present the scheme with both the proposed feature extraction method and KELM to obtain remarkable effects in speed and accuracy compared with other methodologies.

The rest of this paper is organized as follows: Section 2 introduces the details of the machine-learning model for E-tongues, including feature extraction and recognition approaches. The experimental results and analysis are presented in Section 3. Finally, Section 4 concludes this paper.

## 2. Methods

### 2.1. Notations

In this paper, $X={\left[x_{1,}x_{2},\ldots ,x_{m}\right]}^{T}\in R^{m\times d}$ represents the raw responses of one sample, where *m* represents the number of sensors while *d* represents the time length of a sensor response. Here, *d* is decided by the ratio of the sampling time to sampling rate.

### 2.2. Machine Learning Model

In this subsection, our target is to explore a proper machine-learning model for E-tongue recognition on raw sensor responses. Then, the proposed model should have the following properties:

(1) Recognition results should be quickly obtained from a large number of digital pulse-like signals.
(2) Feature specificity should be enhanced by eliminating the common-mode components.
(3) The nonlinear relationships exhibited in sensor responses should be well exploited.

To achieve the above goals, we consequently proposed a machine-learning model for LAPV E-tongues containing a feature specificity enhancement (FSE) scheme and KELM classifier.

#### 2.2.1. Feature Specificity Enhancement

The proposed FSE method abstracts the specificity signals concealed in paired sensor responses by removing the common-mode components in Hilbert space. Thus, the feature extraction method can be formulated as:

(1) $$Z_{ij}^{}=k(x_{i}^{},x_{j}^{}),i\neq j$$

where $x_{i}=\left\{x_{i}^{n}\right\}$ represents the *i*-th sensor response, *n* represents the *n*-th sample, $Z_{ij}=\left\{Z_{ij}^{n}\right\}$ indicates the feature extracted from the *i*-th and *j*-th sensor responses, k(⋅) denotes a kernel function projecting the original specificity component to a nonlinear space. We assumed that an intact sensor response may consist of both specificity and common-mode components. Then, we could redefine $x_{i}^{}$ and $x_{j}^{}$ as:

(2) $$\left\{ \begin{array}{l} x_{i}^{}=x_{c}^{ij}+x_{s}^{ij}\\ x_{j}^{}=x_{c}^{ij}-x_{s}^{ij} \end{array}\right.$$

where $x_{c}^{ij}$ and $x_{s}^{ij}$ denote the common-mode component and the specificity signal in paired sensor responses, respectively. Hence, $x_{c}^{ij}$ and $x_{s}^{ij}$ can be presented as follows:

(3) $$\begin{array}{l} x_{c}^{ij}=\left(x_{i}^{}+x_{j}^{}\right)/2\\ x_{s}^{ij}=\left(x_{i}^{}-x_{j}^{}\right)/2 \end{array}$$

It is obvious that $x_{s}^{ij}$ is the useful portion (specificity part) for further classification while $x_{c}^{ij}$ is the common-mode component containing rare classification information between Sensor *i* and *j*. Thus, we decided to adopt $x_{s}^{ij}$ for following process. Moreover, we introduced the kernel function to deal with the nonlinearity information presented in the specificity signals and solve “dimension disaster” problem of LAPV responses in space projection [31]. In general, some differentiable formulae including polynomials, Gaussian (radial basis function, RBF), exponential, sigmoid and delta functions can be selected as the kernel function. For these kernel functions, RBF causes the lowest computation load due to the minimum number of parameters and demonstrates favorable performance in data classification. Therefore, we chose RBF as the primary exploration kernel function in this paper, which is defined as:

(4) $$k(x_{i},x_{j})=k(2x_{s}^{ij})=\exp(-\frac{{\left\|x_{i}-x_{j}\right\|}_{2}^{2}}{2\sigma^{2}})$$

where exp(•) represents an exponential function, σ is the width of the kernel function and ${\left\|\cdot \right\|}_{2}$ denotes the *l*_2_-norm. From this expression, we could infer that the feature size of each sample is *m*(*m*-1) after feature extraction with RBF. In other words, the size of feature subset is only associated with the electrode number of the sensor array instead of the sensor-response length. In addition, the proposed FSE method can achieve meaningful features in unsupervised manner (without any priori labels). As a result, the proposed FSE is a simple and light calculation approach for feature extraction from high dimensional space. The details of FSE have been summarized in Algorithm 1.

**Algorithm 1.** The FSE Method
**Input:**
The sample data matrix $X\in R^{m\times d}$The kernel function form k(⋅), kernel parameters σ
**Output:**
The feature matrix $Z\in R^{m\times (m-1)}$
**Procedure:**
for i=1,…,m  for j=1,…,m    $Z_{ij}=k(x_{i,}x_{j}),\forall i\neq j$ end  endoutput the feature matrix $Z\in R^{m\times (m-1)}$

#### 2.2.2. Kernel Extreme Learning Machine

The extreme learning machine (ELM) is a fast learning algorithm with a single hidden layered forward neural network [32]. A recent research work reported the feasibility of this method in liquid recognition [33]. It randomly initializes the input weights $W={\left[w_{1},w_{2},\ldots ,w_{L}\right]}^{T}\in R^{L\times D}$ and bias $b=\left[b_{1},b_{2},\ldots ,b_{L}\right]\in R^{L}$. The output weight matrix $\beta \in R^{L\times C}$ is calculated based on the output matrix of the hidden layer analytically. The output matrix H of the hidden layer with *L* hidden neurons is computed as:

(5) $$H={\left[\begin{array}{ccc} g\left(w_{1}^{T}x_{1}+b_{1}\right) & \cdots & g\left(w_{L}^{T}x_{1}+b_{L}\right)\\ \vdots & \ddots & \vdots \\ g\left(w_{1}^{T}x_{N}+b_{1}\right) & \cdots & g\left(w_{L}^{T}x_{N}+b_{L}\right) \end{array}\right]}_{N\times L}$$

where g(⋅) is the activation function and *N* is the number of the training samples. The ELM learning method reaches the tradeoff between minimum training error and smallest norm of weights as follows:

$$\underset{\beta }{\min}\frac{1}{2}{\left\|\beta \right\|}^{2}+\mu \cdot \frac{1}{2}\sum_{i=1}^{N}{\left\|\xi \right\|}^{2}$$

(6) $$s.t.\;h\left(x_{i}\right)\beta =t_{i}-\xi_{i},i=1,2,\ldots ,N$$

where μ is the regularization coefficient, ξ denotes the prediction error on training set. The approximate solutions of output weight matrix β are directly calculated as:

(7) $$\beta =\{\begin{array}{cc} {\left(H^{T}H+\frac{I_{L\times L}}{\mu }\right)}^{-1}H^{T}T, & N\geq L\\ H^{T}{\left(H^{T}H+\frac{I_{N\times N}}{\mu }\right)}^{-1}T, & N<L \end{array}$$

where $T={\left[t_{1},t_{2},\ldots ,t_{N}\right]}^{T}\in R^{N\times C}$. Therefore, the final output can be computed as:

(8) $$f\left(x\right)=h\left(x\right)H^{T}{\left(H^{T}H+\frac{I_{N\times N}}{u}\right)}^{-1}T$$

The KELM introduces a kernel function when calculating the output of the network [34] which denotes $K_{ij}=h\left(x_{i}\right)\cdot h\left(x_{j}\right)=k\left(x_{i},x_{j}\right)$, thus, Equation (17) can be expressed by:

(9) $$f\left(x\right)=\left[\begin{array}{c} k\left(x,x_{1}\right)\\ \vdots \\ k\left(x,x_{N}\right) \end{array}\right]{\left(K+\frac{I_{N\times N}}{\mu }\right)}^{-1}T$$

When the kernel function is employed by KELM, not only is the computation time reduced, but also the number of the hidden layer is free of pre-designation.

## 3. Experiments and Discussions

### 3.1. Datasets

In this section, two experimental datasets about LAPV-based E-tongues were conducted to verify the effectiveness of the proposed machine learning model. One dataset was collected from the E-tongue we designed while the other one was a public E-tongue benchmark.

#### 3.1.1. Our Own Dataset

**Figure 1:**
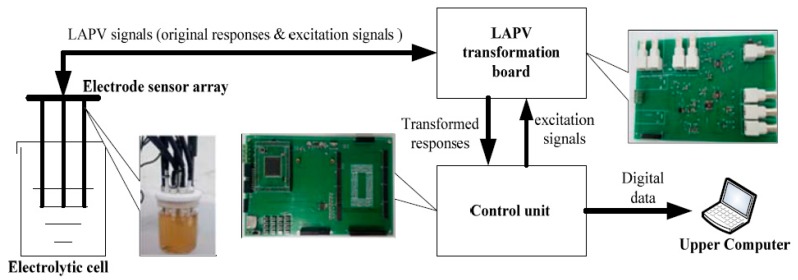
Structure of developed large amplitude pulse voltammetry (LAPV) electronic tongue (E-tongue).

This dataset was acquired from a self-designed E-tongue system, as shown in Figure 1. The system is composed of an electrolytic cell, a sensor array, a signal transformation board, a control unit and an upper computer [35]. The sensor array comprises one reference electrode, one auxiliary electrode and six working electrodes to form a three-electrode system on the basis of multiple LAPV (MLAPV) [28]. A MLAPV-type E-tongue combines the excitation pulses of different potential intensities, so that the solution composition with different properties can show unique oxidation, charging and discharging characteristics at different pulse-like excitation potentials. Meanwhile, the excitation pulse interval is used as the frequency parameter, which makes corresponding responses of working electrodes show special electrochemical characteristics in different frequency segments. As a result, the overall characteristics of solution compositions can be sampled at multi-frequency scale, enriching the feature information for further identification [36].

The working electrodes include gold, platinum, palladium, titanium, tungsten and silver types. The auxiliary and reference electrodes are made of pillar platinum and Ag/AgCl solution, respectively. Before each measurement, the working and auxiliary electrodes were cleaned with a polishing cloth and polishing powder to make the surface mirror-polished, while the reference electrode was washed with distilled water. After each measurement, all the electrodes were washed with distilled water to prevent the residues adhesion.

Seven beverages including red wine, white spirit, beer, oolong tea, black tea, maofeng tea and pu’er tea were selected for experiments. With regard to each type of tea, 2 g of solid tea leaves weresoaked with 200 mL of distilled water for 5 min. Afterwards, the original solution of tea can be attained by filtering out the liquid, while the ones of red wine, white spirit and beer were bought directly from the manufacturers. Then, we formulated samples at different concentrations with both the original solution and distilled water which maintained the temperature around 25 °C. Accordingly, low, medium and high concentration samples were formulated for each beverage according to the ratio of original solution at 14%, 25% and 100%, respectively. We tested each prepared sample three times in an hour with the designed E-tongue. In total, 63 (7 kinds × 3 concentrations × 3 times) samples were collected for seven kinds of liquid.

**Figure 2:**
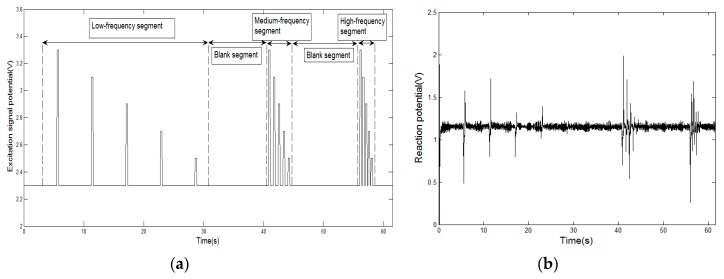
(**a**) Excitation signal of multiple LAPV (MLAPV). (**b**) Typical response of a working electrode.

The excitation signal of MLAPV includes three frequency segments, 0.2 Hz (low frequency), 1 Hz (medium frequency) and 2 Hz (high frequency), in one-time cycle to stimulate different transient pulse-like responses. As Figure 2a shows, the excitation signal on the auxiliary electrode lasts 60 s per circle. A blank segment is located between two frequency segments as a cooling-off period for electrodes. While, each frequency segment contains five pulses with voltages of 3.3, 3.1, 2.9, 2.7 and 2.5 V relative to a reference voltage of 2.3 V. In our circuit design, the signal potential acting on the solution was equal to the difference between the pulse voltage and the reference voltage. Thus, the practical pulse voltages in the solution are 1.0, 0.8, 0.6, 0.4 and 0.2 V, respectively. Subsequently, the response on a working electrode, as shown in Figure 2b, is a series of transient signal by the excitation signal. Totally, 9000 response points have been collected for one working electrode in terms of the sampling rate at 150 Hz. As a result, a 6 × 9000 data matrix can be obtained for one experiment owing to six working electrodes.

#### 3.1.2. Public Benchmark

In order to evaluate the effectiveness of the proposed method comprehensively, a benchmark dataset of 13 kinds of liquid collected on the basis of LAPV E-tongue was used [33]. The 13 kinds of liquid were beer, red wine, white spirit, black tea, Mao Feng tea, Pu’er tea, oolong tea, coffee, milk, cola, vinegar, medicine and salt. The dataset consisted of 114 samples and the matrix size of each sample was 6 × 2050. This corresponds to six sensors and 2050 points collected per sensor. In this paper, we used this dataset with fourfold cross-validation strategy.

### 3.2. Method Settings

In order to evaluate the proposed machine learning model comprehensively, we chose several popular feature extraction methods and classifiers to form different machine-learning models for comparisons.

For feature extraction methods, we adopted Raw Data (RD), PCA, DWT and the proposed FSE approaches for the following evaluation. Here, RD means using raw responses directly for recognition [37]. PCA effectively removes the redundant components from the original data based on orthogonal transformation by keeping the components with the large variance [38]. DWT can process MLAPV signals by employing class-separability criterion to find the suitable level for wavelet decomposition [30]. For fair comparison, we defined another kernel-based method (KBM) using the same kernel functions and parameters of FSE with common-mode signals. The formulation of KBM is by:

(10) $$Z_{i}=\exp(-\frac{{\left\|x_{i}\right\|}_{2}^{2}}{2\sigma^{2}}),i=1,\ldots ,m$$

In terms of the recognition part, Support Vector Machine (SVM) [31], Random Forest (RF) [39], Linear Discriminant Analysis (LDA) [40] and Naive Bayes (NB) [41] were considered in addition to KELM. We selected SVM as a representative of the classifier in a tradeoff between approximation and generalization. Moreover, RF is a typical method using the classifier-ensemble strategy. SVM can employ different kernel functions to map low-dimensional samples to high-dimensional spaces for more accurate classification. We selected RBF as the kernel function of SVM to perform nonlinear projection. After grid optimization, the penalty coefficient C and kernel parameter of SVM was set to 1000 and 0.001, respectively. RF has few parameters to be adjusted and it works well on both classification and regression problems. In the tradeoff of computation load and classification accuracy, the decision-tree number of RF is set to 50. The basic idea of LDA is to project high-dimensional samples into an optimal discriminant space where the ratio of inner-class and the inter-class distance is minimized. Additionally, NB is a classification algorithm based on probability theory.

### 3.3. Model Performance Evaluation

As mentioned above, the machine-learning models to be evaluated were composed of different feature extraction methods and classifiers. The available choices of the feature extraction methods are RD, PCA, DWT, KBM and FSE. Meanwhile, the recognition part should be one of these classifiers—RF, LDA, NB, SVM and KELM. Each model was tested on both datasets for recognition rates, running time and memory cost.

In terms of data usage, we adopted leave-one-out (LOO) [42] and four-fold cross validation for our own dataset and the public benchmark, respectively. For our own dataset, we abstract eight of nine samples from each category as training samples, while the remaining samples are treated as validation ones. That is to say, the sizes of training and validation sets were 56 and 7, respectively. Considering that there are nine different ways to select training samples for each kind, we calculated the average accuracy and associate standard deviation (STD) of these nine recognitions as final scores of a certain model. As for the public benchmark, we divided overall 114 samples into four groups (28, 29, 28 and 29 samples, respectively), and ensured that each group covered all the categories. Then, three of four groups were utilized as training data while the remaining one was used as validation data. At last, the average accuracy and associate STD from these four different recognitions were seemed as a score of a certain model.

Table 1 illustrates the average accuracies and associate STD of different models. As for our own dataset, FSE-KELM obtains the highest accuracy (95.24%), and FSE-SVM reaches the second place. FSE-RF is the best model among the RF-based models, showing a steady recognition performance fluctuating in the range of 61% to 81%. When the RD features are input to LDA and NB, we cannot obtain any outputs due to memory overflow caused by big feature size. Compared with other three classifiers, both LDA and NB have poor performance. 79.37% and 68.25% are the highest values among the LDA’s and NB’s accuracies, respectively. Both SVM and KELM have dramatic decline in accuracy when coupled with RD, which may be caused by the high dimension of features. We noticed that the recognition rates of the SVM-based models exceeded the KELM ones when using RD, PCA and KBM as feature extraction approaches. Considering the public benchmark, FSE-KELM and FSE-SVM still hold the first and second place in recognition, which improve by nearly 38% and 70% over RD-KELM and RD-SVM, respectively. Furthermore, FSE-LDA follows with an accuracy of 79.80%, though LDA cannot work coupled with RD. It is apparent that the models with KBM have poor performance owing to the impact of the common-mode components. Considering the STD of accuracy, FSE performs robustly enough, with the STD of FSE varying from 4.0% to 15.22%. It also obtained the lowest value of 7.10%, 7.10% and 6.73% with LDA, SVM and KELM, respectively, among the feature extraction methods, in our own dataset, respectively. Other STD values of FSE are still in a moderate range, although not the lowest. Generally, we can infer some universal similarities from the recognition rates in Table 1:

(1) The proposed FSE method is an effective feature extraction method in E-tongue identifications. FSE based models are the winners on accuracy in all scenarios with the same classifiers.
(2) The recognition rates of the KBM-based models seriously fall for the public benchmark, which indicates the importance of common-mode signal reduction. We believe FSE method enhances the feature specificity via common-mode signal counteraction.
(3) As for classifiers, KELM-based machine-learning models reached the highest accuracy six times in 10 scenarios (2 datasets × 5 feature extraction methods). This means that KELM has sufficient approximation and generalization ability for E-tongue recognition.

### 3.4. Time and Memory Cost Comparisons

In the view of execution time, less time implies a lower energy consumption and faster detection speed for E-tongue devices. Table 2 provides the execution time of different machine-learning models. Almost the same trends have appeared on both our own dataset and the public benchmark. The consumption time of the models with KBM and FSE seems obviously shorter (two orders of magnitude in most cases) than those of other models. Although the time of KBM is slightly less than that of FSE, the accuracies of KBM-based models are unstable. When using SVM, FSE is nearly 40 times faster than RD on our own dataset and 50 times faster than DWT on the public benchmark. Hence, FSE is the most suitable feature extraction method considering both accuracy and time consumption. Moreover, KELM expends the least execution time in most cases (seven out of 10 scenarios) among all the classifiers. This proves that KELM requires little computation as well as providing excellent classification results.

**Figure 3:**
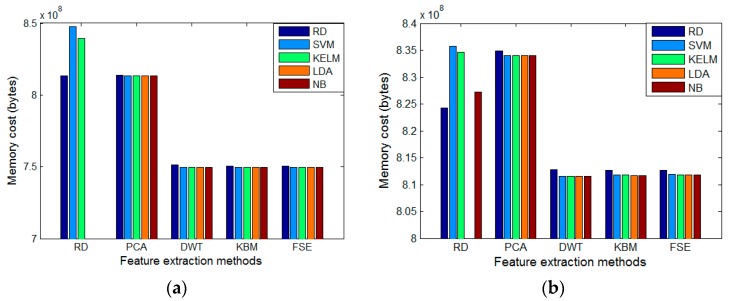
Memory cost: (**a**) Our own dataset. (**b**) Public benchmark.

In addition, the details of memory cost are shown in Figure 3. Regarding memory consumption, RD and PCA are significantly higher than the other three methods. When SVM is combined with RD, the memory consumption is the highest, and for other feature extraction methods, the RF has the highest memory cost compared to the other four classifiers. It is noted that when the original feature is used, the LDA and NB classifiers cannot work because the feature dimension is too high, so there is no record of the two classifiers at this time.

### 3.5. Parameter Sensitivity Analysis

#### 3.5.1. Analysis of FSE Parameters

**Figure 4:**
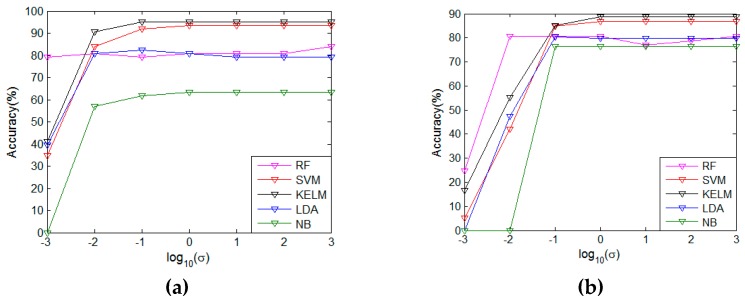
Sensitivity analysis of feature specificity enhancement (FSE) (σ): (**a**) Our own dataset. (**b**) Public benchmark.

The kernel function width σ is an adjustable parameter in the proposed FSE method. In order to observe the parameter sensitivity of FSE, we tuned σ from the parameter set $\left\{10^{-3}{,10}^{-2},\cdots {,10}^{3}\right\}$ and collected the accuracy on both two datasets. The results are drawn in Figure 4a and b. In particular, NB cannot work for our own datasets on $\sigma =10^{-3}$ and the benchmark on $\sigma =10^{-2}$, because parts of the features are equal to 0. For our own dataset, as shown in Figure 4a, FSE-RF is insensitive to the alteration of the width. FSE, coupled with the other four classifiers, has the same tendency in that the accuracy moves upwards rapidly in the scale $\sigma ={[10}^{-3}{,10}^{-1}]$ and then keeps the performance steady in the follow-up scope. In terms of the public benchmark, as shown in Figure 4b, all the FSE-based methods have an early rising stage and, then, stable curves would be presented in the subsequent range $\sigma ={[10}^{-1}{,10}^{3}]$. In a word, FSE method has strong robustness for parameter variation. In practice, when the kernel function width σ belongs to [10^−1^,10^3^], the recognition results are not sensitive to the parameter values.

#### 3.5.2. Analysis of KELM Parameters

**Figure 5:**
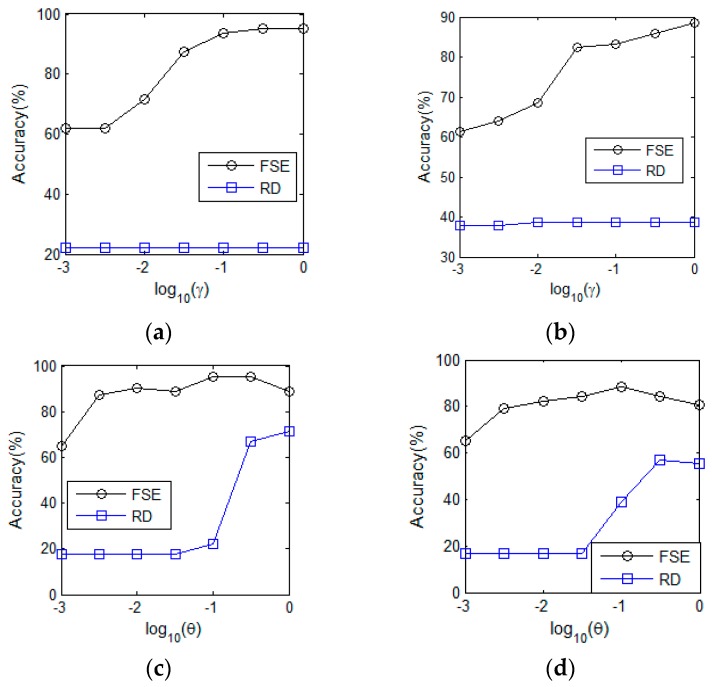
Sensitivity analysis of the kernel extreme learning machine (KELM): (**a**) γ on our own dataset. (**b**) γ on public benchmark. (**c**) θ on our own dataset. (**d**) θ on public benchmark.

The regularization coefficient γ and kernel function width θ are two adjustable parameters in KELM. For better sight of the parameter sensitivity of KELM, we tuned two parameters from the parameter set $\left\{10^{-3}{,10}^{-2},\cdots {,10}^{3}\right\}$. The results of regularization coefficient γ on both our own dataset and the public benchmark are presented in the Figure 5a and b, from which the optimal γ is clearly observed. Thus, γ=1000 was empirically set. Figure 5c and d show the results in terms of kernel function width θ on both datasets, and we have observed that the favorable performance could be obtained when θ=10.

## 4. Conclusions

In this study, we have proposed a machine-learning model for E-tongue recognitions. In the proposed model, FSE method is designed for nonlinear feature extraction while KELM is adopted to accelerate the calculation speed of the model. In experimental comparison, the proposed FSE coupled with KELM achieves both the best accuracy and computational efficiency on the two datasets. It can be summarized that the common-mode signal counteraction of E-tongue sensors and nonlinear projection can make great contribution to E-tongue classification. Meanwhile, KELM shows dominant specialties in data approximation, model generalization and computational-time reduction. We discover that FSE seems to be effective in dealing with high dimensional data, especially for LAPV signals. Moreover, KELM can greatly promote the overall performance in accuracy and speed for E-tongue-based recognitions.

## Figures and Tables

**Table 1:** Accuracy and standard deviation of models (%).

	Classifier	Feature Extraction Methods									
		RD		PCA		DWT		KBM		FSE	
		Acc.	STD	Acc.	STD	Acc.	STD	Acc.	STD	Acc.	STD
**Our own dataset**	RF	74.60	17.82	61.90	17.53	77.78	7.10	71.43	15.06	80.95	13.47
	LDA	-	-	46.03	16.19	53.97	16.19	60.32	17.53	79.37	7.10
	NB	-	-	46.03	17.53	55.56	14.20	68.25	16.19	63.49	15.22
	SVM	34.92	11.88	80.95	15.06	66.67	16.50	71.43	17.82	93.65	7.10
	KELM	22.22	9.78	69.84	21.76	93.65	9.78	68.25	22.11	95.24	6.73
**Public benchmark**	RF	71.59	6.85	70.32	7.91	75.38	5.28	18.27	6.07	79.63	6.07
	LDA	-	-	61.63	10.26	63.89	5.61	24.53	3.89	79.80	11.90
	NB	59.28	12.11	63.38	9.19	66.70	3.17	24.38	8.25	76.34	8.04
	SVM	16.76	2.14	60.66	5.62	69.36	3.85	24.53	3.89	86.73	4.00
	KELM	50.93	3.61	72.67	11.31	78.76	4.40	29.88	3.06	88.65	4.36

**Table 2:** Time consumption of models.

	Classifier	Feature Extraction Methods				
		RD	PCA	DWT	KBM	FSE
**Our Own Dataset**	RF	164.74 s	37.80 s	52.01 s	4.32 s	4.18s
	LDA	-	39.61 s	53.71 s	1.03 s	1.65 s
	NB	-	38.31 s	50.84 s	0.45 s	0.53 s
	SVM	10.64 s	33.31 s	47.92 s	0.30 s	0.45 s
	KELM	6.40 s	37.50 s	53.77 s	0.27 s	0.31 s
**Public Benchmark**	RF	25.08 s	11.19 s	97.98 s	2.26 s	2.62 s
	LDA	-	9.16 s	91.28 s	0.78 s	1.67 s
	NB	3.34 s	8.77 s	90.62 s	0.25 s	0.33 s
	SVM	2.56 s	8.33 s	89.45 s	0.11 s	0.17 s
	KELM	0.95 s	7.89 s	90.32 s	0.09 s	0.16 s
